# High temperature ameliorates high-fat diet-induced obesity by promoting ceramide breakdown in skeletal muscle tissue

**DOI:** 10.1093/lifemeta/loae012

**Published:** 2024-04-03

**Authors:** Qiankun Wang, Lupeng Chen, Junzhi Zhang, Yue Liu, Yi Jin, Jian Wu, Zhuqing Ren

**Affiliations:** Key Laboratory of Agriculture Animal Genetics, Breeding and Reproduction of the Ministry of Education, College of Animal Science and Technology, Huazhong Agricultural University, Wuhan, Hubei 430070, China; College of Animal Science and Technology, Henan University of Science and Technology, Luoyang, Henan 471000, China; Key Laboratory of Agriculture Animal Genetics, Breeding and Reproduction of the Ministry of Education, College of Animal Science and Technology, Huazhong Agricultural University, Wuhan, Hubei 430070, China; Key Laboratory of Agriculture Animal Genetics, Breeding and Reproduction of the Ministry of Education, College of Animal Science and Technology, Huazhong Agricultural University, Wuhan, Hubei 430070, China; Key Laboratory of Agriculture Animal Genetics, Breeding and Reproduction of the Ministry of Education, College of Animal Science and Technology, Huazhong Agricultural University, Wuhan, Hubei 430070, China; Key Laboratory of Agriculture Animal Genetics, Breeding and Reproduction of the Ministry of Education, College of Animal Science and Technology, Huazhong Agricultural University, Wuhan, Hubei 430070, China; Key Laboratory of Agriculture Animal Genetics, Breeding and Reproduction of the Ministry of Education, College of Animal Science and Technology, Huazhong Agricultural University, Wuhan, Hubei 430070, China; Key Laboratory of Agriculture Animal Genetics, Breeding and Reproduction of the Ministry of Education, College of Animal Science and Technology, Huazhong Agricultural University, Wuhan, Hubei 430070, China; Frontiers Science Center for Animal Breeding and Sustainable Production, Wuhan, Hubei 430070, China; Hubei Hongshan Laboratory, Wuhan, Hubei 430070, China

**Keywords:** high temperature, obesity, insulin resistance, ceramide breakdown, lipid droplets

## Abstract

Obesity is considered an epidemic often accompanied by insulin resistance (IR). Heat treatment (HT) has been shown to prevent high-fat diet-induced IR in skeletal muscle, but the underlying mechanisms are poorly understood. In this study, we discovered that high temperature alleviated the hallmarks of obesity by promoting glycogen synthesis and lowering blood glucose levels in skeletal muscle tissue (SMT). Additionally, HT maintained the decay phase of heat shock factor 1 (HSF1), leading to the activation of gene expression of heat shock proteins (HSPs), which contributed to the alleviation of IR in SMT of diet-induced obese (DIO) mice. Metabolomics and lipidomics analyses showed that HT promoted ceramide (Cer) breakdown, resulting in an elevation of both sphingomyelin (SM) and sphingosine, which further contributed to the amelioration of IR in SMT of DIO mice. Importantly, the increase in sphingosine was attributed to the heightened expression of the acid ceramidase N-acylsphingosine amidohydrolase 1 (ASAH1), and the inhibition of ASAH1 attenuated HT-relieved IR in SMT of DIO mice. Surprisingly, high temperature increased the composition of Cer and cholesteryl ester in lipid droplets of skeletal muscle cells. This not only helped alleviate IR but also prevented lipotoxicity in SMT of DIO mice. These findings revealed a previously unknown connection between a high-temperature environment and sphingolipid metabolism in obesity, suggesting that high temperature can improve IR by promoting Cer catabolism in SMT of obese mice.

## Introduction

In the context of improved living standards, obesity has emerged as a chronic metabolic disease with global implications, contributing to a range of adverse outcomes [1]. For instance, it may result in insulin resistance (IR), thereby negatively impacting individual health and burdening healthcare systems as a whole [2]. The skeletal muscle tissue (SMT) plays a crucial role in systemic insulin-mediated glucose uptake, and reduced insulin-stimulated glucose uptake is primarily caused by IR [3, 4]. IR is a key factor in the development of type 2 diabetes and is closely associated with obesity [5, 6]. Moreover, the accumulation of intramyocellular lipids impairs insulin sensitivity. For instance, insulin sensitivity is negatively correlated with skeletal muscle triglyceride content and depends on the fatty acid composition [7–9]. These findings suggest that targeting IR in SMT as a means to address obesity may be an effective strategy, although many questions remain unanswered. Obesity is classified into physiological or metabolic types [10, 11]. Metabolic obesity, often induced by high-fat diet (HFD), is frequently accompanied by elevated blood glucose and accumulation of ceramide (Cer) in skeletal muscles [12]. Furthermore, genomic and lipidomic studies have shed light on the role of sphingolipids in various physiological processes and as crucial regulators of cellular stress. Cer is a product of sphingolipid metabolism and can also act as a second messenger to regulate sphingolipid signaling [13]. Cer is synthesized by hydrolyzing sphingolipids from lysosomes and endosomes, or *de novo* synthesis from fatty acids, serine, and palmitoyl-CoA [14]. In addition, HFD-induced Cer accumulation increases the mitochondrial burden, directly contributing to IR in the SMT [4, 15]. Conversely, reducing Cer accumulation has been shown to improve IR in diet-induced obese (DIO) mice.

Several recent studies have highlighted the significance of heat shock proteins (HSPs) in mitigating obesity-induced IR. For instance, heat treatment (HT) has been shown to upregulate HSP72 and HSP25, which effectively inhibit the activation of c-Jun NH_2_-terminal kinase (JNK) and inhibitor of nuclear factor-κB (NF-κB) kinase (IKK-β), respectively, ultimately protecting skeletal muscle from HFD-induced IR [16]. Moreover, increasing intracellular levels of HSP70, both within skeletal muscle cells and in circulation, have been found to alleviate IR [17]. Intriguingly, thermotherapy, which involves the use of heating methods like saunas and hot baths to induce temporary hyperthermia, has gained attention as a potential strategy for managing metabolic diseases [18–20]. Furthermore, heat shock factor 1 (HSF1) is notably reduced in the SMT of obese mice when compared to normal mice [21]. The overexpression of HSF1 in the muscle increases insulin sensitivity in the SMT. However, the precise mechanisms through which HT prevents IR remain largely unexplored.

The primary objective of this study was to investigate whether acute HT can effectively prevent IR in the SMT of DIO mice. Additionally, we aimed to shed light on the underlying mechanism by which HT alleviated IR in the SMT of DIO mice. We put forth the hypothesis that HT-induced HSP70 promoted the reprogramming of sphingolipid metabolism in the SMT, leading to an improvement in IR among DIO mice. Our findings indicated that HT indeed facilitates the interaction between HSP70 and HSF1, thereby activating sphingolipid metabolism and promoting Cer breakdown. As a result, this process attenuates heat-induced injury in DIO mice and ultimately alleviates IR in the SMT of DIO mice.

## Results

### Mitigation of heat stress damage to SMT by HT in DIO mice

To investigate the impact of HT on the heat stress response in the SMT of both normal and DIO mice, we examined the levels of HSP70, reactive oxygen species (ROS), and apoptosis-related proteins. Our results demonstrated that HT increased the expression of HSP70 in the SMT of both normal and DIO mice (Supplementary Fig. S1a−c, e, and f). Interestingly, while HT led to elevated ROS levels in the SMT of normal mice, it did not affect ROS levels in the SMT of obese mice (Supplementary Fig. S1d). Furthermore, we observed that HT caused an increase in the concentrations of apoptosis-related proteins, including cytochrome C (CytoC) and tumor necrosis factor receptor 1 (TNFR1), in the SMT of normal mice, whereas these protein levels remained unchanged in the SMT of obese mice (Supplementary Fig. S1e, g, and h). These findings suggested that HT could induce damage to the SMT in normal mice, while DIO mice were able to mitigate heat stress damage to the SMT caused by HT.

### HT improves carbohydrate and lipid metabolism and insulin sensitivity in the SMT of DIO mice

**Figure 1:**
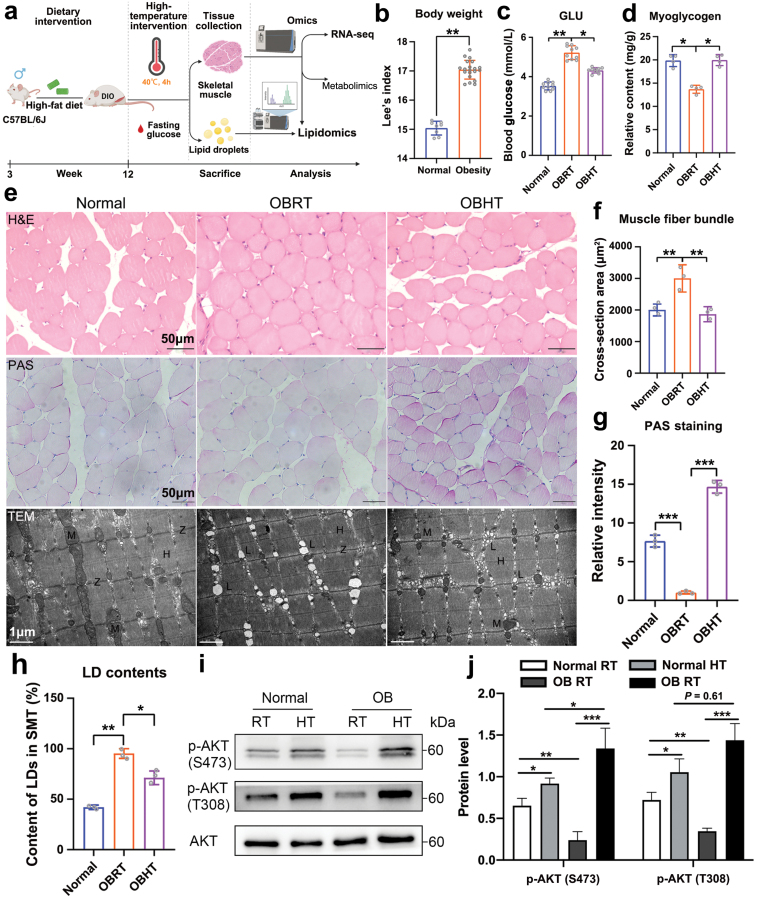
HT promotes myoglycogen synthesis and lipolysis of the SMT in DIO mice. (a) Flow diagram demonstrating the HT experiment on DIO mice. DIO: diet-induced obesity. (b) Lee’s index in obese mice. Normal, standard rat chow-fed mice. ^**^P < 0.01. (c) The level of serum glucose in normal, OBRT, and OBHT groups. *n* = 10. ^*^P < 0.05, ^**^P < 0.01. (d) The level of myoglycogen in the SMT of normal, OBRT, and OBHT groups, *n* = 4. ^*^P < 0.05. (e−h) Histomorphological observation of the SMT in normal, OBRT, and OBHT groups. The top row is hematoxylin and eosin (H&E) staining of the SMT (e, top row) and the statistical analysis of muscle fiber bundles (f). Scale bar = 50 μm. *n* = 3. ^*^P < 0.05, ^**^P < 0.01. The middle row is PAS staining of SMT myoglycogen (e, middle row) and the statistical analysis of myoglycogen (g). Scale bar = 50 μm. *n *= 3. ^***^P < 0.001. The bottom row is TEM results of SMT (e, bottom row) and its LD content statistics (h). TEM, transmission electron microscopy; L, lipid droplets; M, mitochondria; Z, Z line; H, H line. Scale bar = 1 μm. *n* = 3. ^*^P < 0.05, ^**^P < 0.01. (i and j) WB analysis of AKT (S473 and T308) phosphorylation status relative to total AKT in the SMT. *n* = 3. ^*^P < 0.05, ^**^P < 0.01, ^***^P < 0.001.

To investigate the effect of high temperature on carbohydrate and lipid metabolism of the SMT in DIO mice, we performed HT (40°C, 4 h) experiments on DIO mice (Fig. 1a and b). Before hyperthermia, HFD-induced obesity not only increased the size and number of lipid droplets (LDs) in skeletal muscle cells (Fig. 1e) but also elevated the serum glucose levels (Fig. 1c), contributing to the hypertrophy of skeletal muscle fiber bundles in DIO mice (Fig. 1f). Furthermore, we observed a decrease in myoglycogen levels in the SMT of DIO mice following the HFD (Fig. 1d), suggesting the presence of IR in the SMT of HFD-induced obese mice. Interestingly, HT not only decreased the serum glucose level of DIO mice (Fig. 1c) but also increased the myoglycogen content in their SMT (Fig. 1d and g). In addition, HT ameliorated the hypertrophied muscle fiber bundles (Fig. 1f). Transmission electron microscopy (TEM) observations of skeletal muscle revealed that HT reduced the size and content of LDs in DIO mice, indicating that HT promoted lipid utilization in the skeletal muscle of DIO mice (Fig. 1e and h). Furthermore, the phosphorylation levels of AKT473 and AKT308 in the SMT of DIO mice were significantly decreased when compared to that of normal mice (Fig. 1i and j), indicating the presence of IR. HT significantly enhanced the phosphorylation levels of AKT473 and AKT308 in the SMT of both normal and obese mice (Fig. 1i and j). Notably, under HT conditions, the phosphorylation levels of AKT473 and AKT308 in the SMT of obese mice was significantly higher than that in normal mice (Fig. 1i and j), indicating that HT ameliorated IR in the SMT of DIO mice. In summary, these results suggested that HT improves glucolipid metabolism and insulin sensitivity in the SMT of DIO mice.

### HT improves the gene expression of carbohydrate and lipid metabolism in the SMT of DIO mice

**Figure 2:**
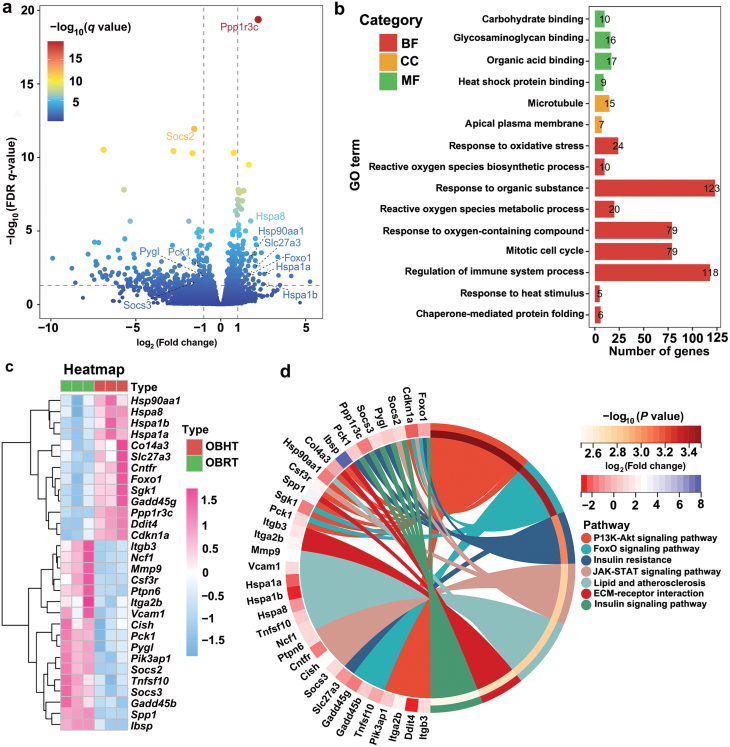
HT affects the transcriptome profiles of the SMT in DIO mice. (a) Volcano plot of DEGs in the SMT of DIO mice. Upregulated gene: |log_2_(fold change)| > 1, downregulated gene: |log_2_(fold change)| < −1. The top 11 genes are shown. (b) GO enrichment analysis for DEGs in the SMT of DIO mice. BP, biological process; CC, cell component; MF, molecular function. (c) Heatmap of the top 30 DEGs sorted by basemean from the highest to the lowest. (d) KEGG enrichment analysis for the top 30 DEGs in the SMT of DIO mice.

To investigate the mechanism underlying the improvement of carbohydrate and lipid metabolism in the SMT of DIO mice after HT, we conducted RNA sequencing (RNA-seq) analysis of the SMT. The significance criteria used were |log_2_(fold change)| > 1 and *P*adj < 0.05, resulting in the identification of 265 differentially expressed genes (DEGs), including 83 upregulated genes and 182 downregulated genes (Fig. 2a; Supplementary Table S1). Gene Ontology (GO) analysis revealed that HT activated the response of the SMT in DIO mice to heat stimulation, oxidative stress, and oxygenated compounds, and affected HSPs, carbohydrates, glycosaminoglycan binding, and the immune system (Fig. 2b).

By analyzing the expression patterns of the DEGs, we identified 11 genes involved in the regulation of carbohydrate and lipid metabolism, including seven upregulated genes (*Ppp1r3c*, *Hspa8*, *Hsp90aa1*, *Slc27a3*, *FoxO1*, *Hspa1a*, and *Hspa1b*) and 4 downregulated genes (*Socs2*, *Pygl*, *Pck1*, and *Socs3*). This suggested that HT dynamically regulated the process of carbohydrate and lipid metabolism in the SMT of DIO mice. Additionally, a drop-down sorting of basemean was performed to identify the top 30 DEGs from the transcriptome data, including 13 upregulated genes and 17 downregulated genes. Subsequently, Kyoto Encyclopedia of Gene and Genomes (KEGG) pathway enrichment analysis was conducted (Fig. 2c). The analysis revealed that HT affected metabolic processes related to the phosphatidylinositol 3-kinase−protein kinase B (PI3K-AKT) signaling pathway, forkhead box protein O (FoxO) signaling pathway, IR, Janus kinase-signal transducer and activator of transcription (JAK-STAT) signaling pathway, lipid and atherosclerosis, extracellular matrix (ECM) receptor interaction, and insulin signaling pathway (Fig. 2d; Supplementary Fig. S2).

Our findings indicated that HT promoted the expression of genes related to glycogen synthesis (*Ppp1r3c* and *FoxO1*) and suppressed the expression of genes associated with glycogen catabolism (*Socs3*, *Socs2*, *Pck1*, and *Pygl*). This suggested that HT increased the sensitivity of the SMT to insulin signaling and enhanced glucose metabolism in obese mice (Supplementary Fig. S2). Moreover, HT altered the expression of genes related to the PI3K-AKT signaling pathway (Supplementary Fig. S2), which plays a role in regulating glucolipid metabolism in skeletal muscle cells of obese mice [22]. Overall, our results suggested that HT may improve insulin sensitivity in the SMT by inducing the reprogramming of carbohydrate and lipid metabolism in DIO mice.

### HT improves carbohydrate and lipid metabolism in the SMT of DIO mice depending on HSF1

**Figure 3:**
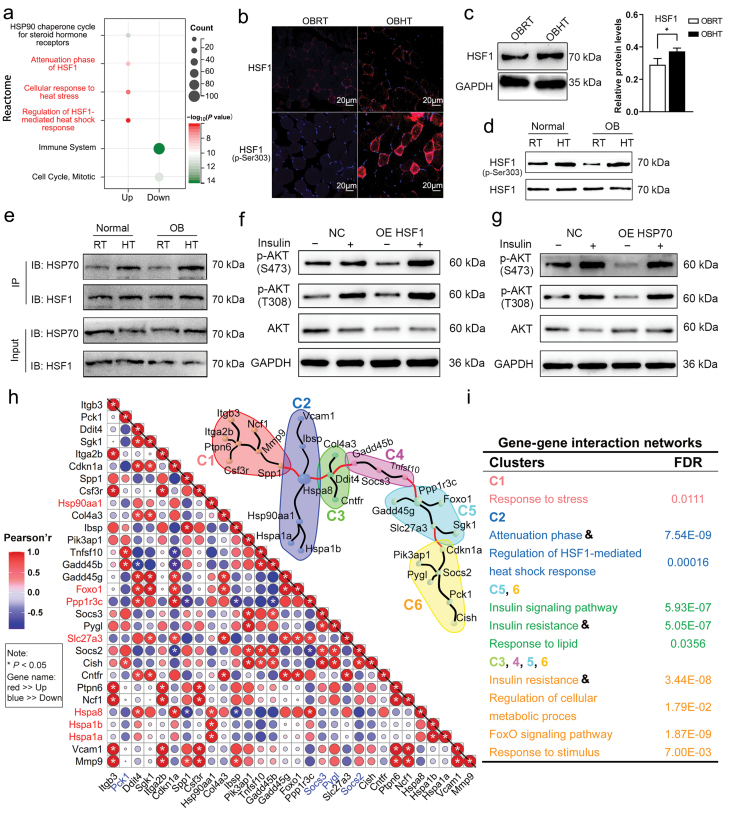
HT keeps the attenuation phase of HSF1 in the SMT of DIO mice. (a) The most enriched reactomes of DEGs in the SMT of DIO mice. (b) Immunofluorescence staining of HSF1 and HSF1 (p-Ser303) protein in OBRT and OBHT groups after HT. Red is positive signaling, and blue is DAPI signaling. (c and d) WB detection of HSF1 (c) and HSF1 (p-Ser303) (d) protein expression in OBRT and OBHT groups. *n* = 3. ^*^P < 0.05, ^***^P < 0.001. (e) CoIP assay detecting the interactions between HSF1 and HSP70 protein. (f) WB analysis of AKT phosphorylation at the S473 and T308 sites in C2C12-differentiated myotubes overexpressing HSF1. (g) WB analysis of AKT phosphorylation at the S473 and T308 sites in C2C12-differentiated myotubes overexpressing HSP70. (h and i) Pearson correlation analysis between the top 30 DEGs (h) and the clustering analysis of the gene-gene interaction network (i). Pearson’r, pearson relation. ^*^P < 0.05. & indicates the most critical cluster analysis among DEGs after HT.

To understand the mechanism by which HT improved carbohydrate and lipid metabolism in mouse SMT, we conducted a functional enrichment analysis of all DEGs at the transcriptome level. The analysis revealed that HT maintained gene expression in the decay phase of HSF1 and promoted HSF1-mediated heat shock response in the SMT of DIO mice (Fig. 3a). Previous studies have shown that phosphorylation of HSF1 enhances its binding to HSPs during the decay phase, leading to increased levels of HSPs in cells [23, 24]. As a result, we sought to investigate the impact of HT on HSF1 and its phosphorylation level. Western blot (WB) analysis revealed that HT enhanced HSF1 expression in obese mice (Fig. 3c). Furthermore, the phosphorylation level of HSF1 at the Ser303 site was notably reduced in obese mice when compared to normal mice under room temperature (RT) conditions (Fig. 3d). While there was no significant difference in the phosphorylation level of HSF1 at the Ser303 site between obese and normal mice under HT, HT did simultaneously elevate the phosphorylation level of HSF1 at the Ser303 site in normal mice when compared to RT groups. This elevation may contribute to the increased expression of HSPs. Furthermore, co-immunoprecipitation (CoIP) data demonstrated that while HT enhanced the interactions between HSF1 and HSP70 in the SMT of both normal and DIO mice, DIO mice exhibited a stronger binding of HSF1 to HSP70 under HT conditions compared to normal mice (Fig. 3e). This suggested that the interactions between HSF1 and HSP70 may play a role in improving insulin sensitivity and carbohydrate and lipid metabolism.

To further investigate the roles of HSF1 and HSP70 in regulating insulin sensitivity and glucolipid metabolism, we individually overexpressed HSF1 and HSP70 in differentiated C2C12 myotubes and examined the phosphorylation levels of AKT473 and AKT308 as well as genes related to glucolipid metabolism. Overexpression of HSF1 promoted the phosphorylation of AKT473 and AKT308 (Fig. 3f; Supplementary Fig. S3a), whereas overexpression of HSP70 increased the phosphorylation of AKT308 without affecting the phosphorylation of AKT473 (Fig. 3g; Supplementary Fig. S3b). Additionally, overexpression of both HSF1 and HSP70 enhanced the mRNA expression of *Glut4*, *Irs-1*, and *Atgl* (Supplementary Fig. S3c and d), indicating the regulatory role of HSF1 and HSP70 in carbohydrate and lipid metabolism.

To determine whether the attenuation of HSF1 by HT was associated with the expression of carbohydrate and lipid metabolism genes in the SMT of DIO mice, we performed Pearson correlation analysis on the top 30 DEGs related to carbohydrate and lipid metabolism. The analysis revealed that HT-activated HSP genes (*Hspa8*, *Hspa1a*, *Hspa1b*, and *Hsp90aa1*) showed a negative correlation with IR-related genes (*Mmp9*, *Ncf1*, *Socs3*, *Socs2*, *Pck1*, and *Pygl*), and a significant positive correlation with genes involved in the insulin signaling pathway (*FoxO1*, *Ppp1r3c*, and others) (Fig. 3h). Furthermore, we observed that *Mmp9* and *Ncf1* were associated with lipid oxidation, indicating that HT may attenuate lipid oxidative damage in the SMT of obese mice (Fig. 3h; Supplementary Fig. S2). Finally, gene–gene interaction network and clustering analysis were performed on the top 30 DEGs in the SMT of DIO mice (Fig. 3h and i). The analysis revealed that HT promoted dynamic regulation of genes involved in gluconeogenesis, glycogen synthesis, and IR (*Socs3*, *Socs2*, *Pck1*, *Pygl*, *FoxO1*, and *Ppp1r3c*), suggesting that HT can inhibit gluconeogenesis and promote myogenic glycogen synthesis by attenuating the phase of HSF1.

### Metabolomics uncovers HT-activated sphingolipid metabolism in the SMT of DIO mice

To investigate the effect of HT on IR in the SMT of DIO mice, we conducted a non-targeted metabolomics analysis. We identified 10,927 fragment ions in the groups subjected to obese RT (OBRT) and obese HT (OBHT) (Supplementary Table S2). Subsequently, we performed discriminant analysis using orthogonal partial least-squares discrimination analysis (OPLS-DA), which showed a clear separation between the groups (*R*^*2*^*Y* = 0.078, *Q*^*2*^ = −0.85) (Supplementary Fig. S4a and b). Using variable important in projection (VIP) > 1 and *P* < 0.05 as screening criteria for differential metabolites (DMs), we identified 107 co-expressed metabolites from the Human Metabolome Database (HMDB) and KEGG databases (Supplementary Table S3). Clustering analysis of these DMs revealed that they primarily consisted of organic heterocyclic compounds, lipids, lipid-like molecules, and organic oxygen compounds (Supplementary Fig. S5a). We observed that HT induced alterations in the majority of SMT metabolites in DIO mice, with 29 organic heterocyclic metabolites showing significant changes (16 increased DMs and 13 decreased DMs), accounting for 30% of all DMs. Notably, HT increased the production of glycerophospholipid and sphingolipid metabolites (Supplementary Fig. S5b), suggesting that it may affect insulin signaling pathways in the SMT of obese mice.

**Figure 4:**
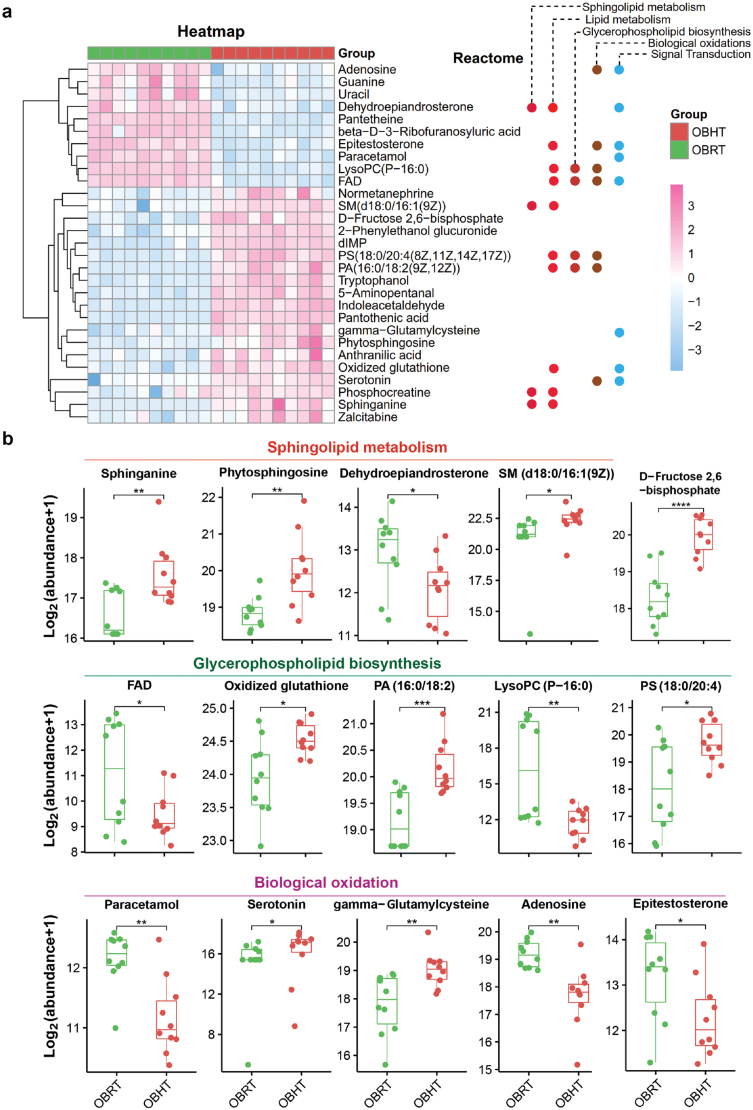
Functional enrichment analysis of the most enriched DMs in the SMT of obese mice. (a) Enriched reactomes involved in 29 DMs of SMT in DIO mice. (b) The effect of HT on sphingolipid metabolism, glycerophospholipid biosynthesis, and biological oxidation in the SMT of DIO mice. SM, sphingomyelin; FAD, flavin adenine dinucleotide; PA, phosphatidic acid; LysoPC, lysophosphatidylcholine. *n* = 10. ^*^P < 0.05, ^**^P < 0.01, ^***^P < 0.001.

To further understand the biochemical processes most affected by HT, we performed a response group enrichment analysis of the 29 DMs in the SMT of DIO mice (Fig. 4a). Among these DMs, 10 were involved in lipid metabolic processes, 8 in signal transduction pathways (with 7 involved in biooxidation), 5 in glycerophospholipid metabolic processes, and 4 in sphingolipid metabolic pathways. We visualized the levels of relevant metabolites involved in sphingolipid metabolism, glycerophospholipid biosynthesis, and biooxidation in the SMT of DIO mice (Fig. 4b). We found that HT activated sphingolipid metabolism by promoting the synthesis of sphingosine, phytosphingosine, and sphingomyelin (SM) (d18:0/16:1(9Z)) in the SMT of DIO mice. HT ultimately disrupted glycerophospholipid biosynthesis by decreasing flavin adenine dinucleotide (FAD) and lysophosphatidylcholine (LysoPC) (P-16:0), and increasing phosphatidic acid (PA) (16:0/18:2) and oxidized glutathione. Additionally, HT enhanced the antioxidant capacity of SMT in DIO mice by increasing the production of serotonin and γ-glutamylcysteine. Importantly, HT promoted the synthesis of D-fructose-2,6-bisphosphate, which may have significant effects on the insulin signaling pathway in the SMT of DIO mice (Fig. 4b).

**Figure 5:**
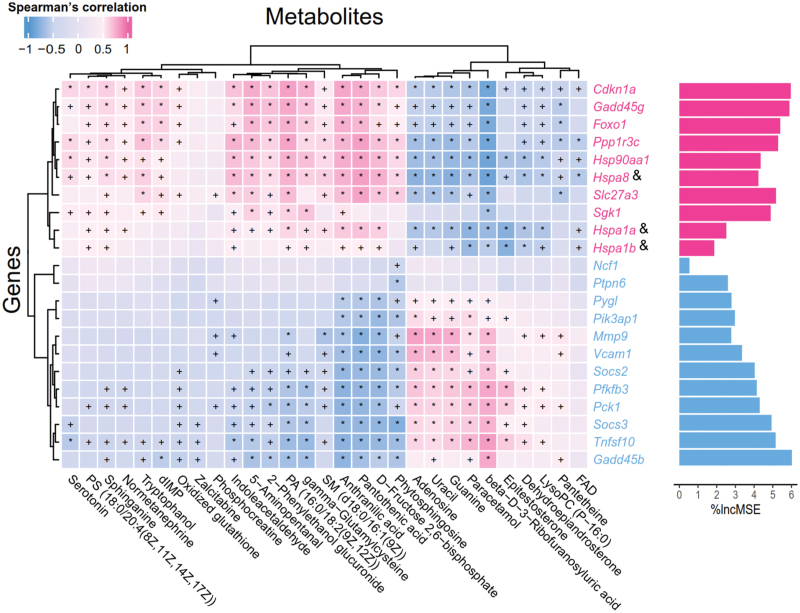
The correlation analysis between DEGs and DMs. Red genes are upregulated, and blue genes are downregulated. & indicates the protein-coding gene of HSP70. +*P* < 0.05, ^*^P < 0.01. Importance was determined based on the percentage increase in mean-squared error (%IncMSE) of metabolite prediction when the relative abundance values of each gene were randomly permuted.

Spearman’s correlation analysis between the DEGs and the DMs further revealed associations between HT-induced genes and sphingolipid metabolism (Fig. 5). There were significant positive correlations between HT-induced genes (*Hspa8, FoxO1, and Ppp1r3c*) and sphingolipids, such as sphinganine, phytosphingosine, and SM (d18:0/16:1(9Z)). The downregulated genes *(Pck1, Socs2,* and *Socs3*) were positively associated with dehydroepiandrosterone. These results implied that HT might regulate the expression of *Hspa8, FoxO1, Ppp1r3c, Pck1, Socs2*, and *Socs3* to modify the sphingolipid metabolism of SMT in DIO mice (Fig. 5).

### HT alters SMT lipid composition in DIO mice

**Figure 6:**
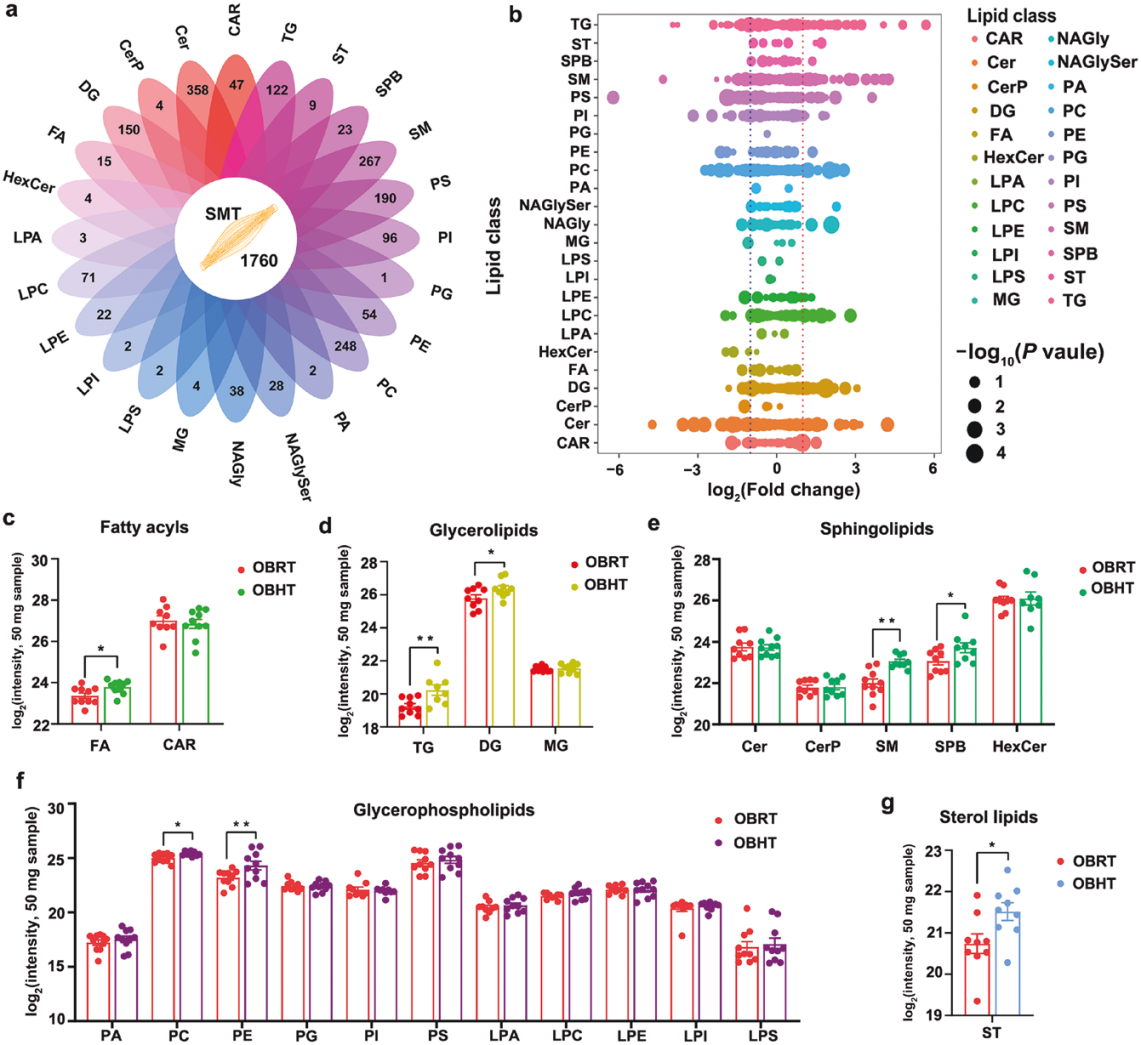
HT affects the lipid composition of the SMT in DIO mice. (a) Flower plot of all significant lipids. (b) The log_2_(fold change) values in lipid class in OBRT vs OBHT group. The significance values are shown as − log_10_(*P* value). (c−g) Effect of HT on fatty acid acyl chains (c), glycerolipids (d), sphingolipids (e), glycerophospholipids (f), and sterol lipids (g) in the SMT of obese mice. FA, fatty acid; DG, diacylglycerol; SM, sphingomyelin; SPB, sphingoid base; PC, phosphatidylcholine; PE, phosphatidylethanolamine; ST, sterol ester. *n* = 10. ^*^P < 0.05, ^**^P < 0.01.

To investigate the effect of HT on lipid metabolism in the SMT of DIO mice, we performed lipidomic analysis after purifying lipids using chloroform/methanol extraction. In the lipidomics analysis, we identified 13,054 fragment ions (Supplementary Table S4), and further identification using the lipid metabolites and pathways strategy (LIPIDMAPS) and LipidBlast databases allowed us to identify 1760 lipids (Fig. 6a; Supplementary Table S5). The identified lipids included 358 Cers, 267 SMs, 248 phosphatidylcholines (PCs), 190 phosphatidylserines (PSs), and 122 triacylglycerols (TAGs). To assess the primary effect of HT on lipid classes in the SMT of DIO mice, we applied screening criteria of *P* < 0.05 and |log_2_(fold change)| > 1 for differential lipids (DLs), resulting in the identification of 59 DLs in the SMT of DIO mice (Fig. 6b; Supplementary Table S5). We classified all the DLs and observed an increase in fatty acyls (Fig. 6c), monoacylglycerols (MGs), and diacylglycerols (DGs) in glycerol esters (Fig. 6d), Cers and sphingoid bases (SPBs) in sphingolipids (Fig. 6e), PCs and phosphatidylethanolamines (PEs) in glycerophospholipids (Fig. 6f), and sterol esters (STs) (Fig. 6g) in the SMT of obese mice after HT. These findings indicated that HT alters the lipid composition of the SMT in DIO mice, leading to changes in fatty acyls, glycerol esters, sphingolipids, glycerophospholipids, and STs.

### HT promotes Cer breakdown in the SMT of DIO mice

**Figure 7:**
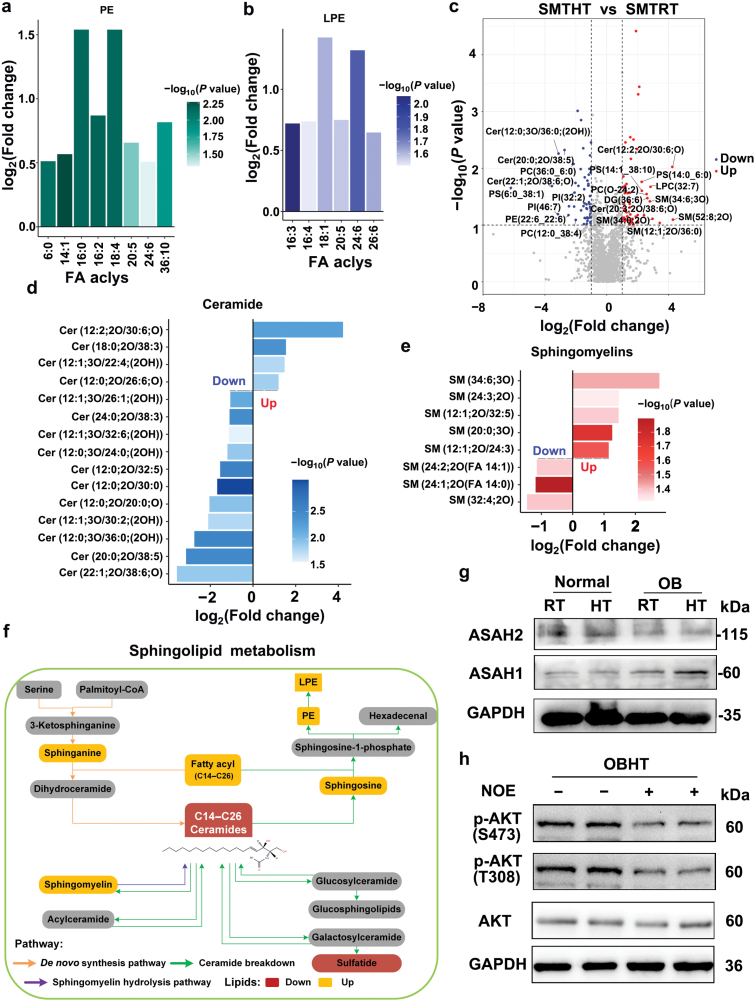
HT promotes Cer breakdown in the SMT of obese mice. (a and b) The intensity of individual fatty acyl chains associated with different PE (a) and LPE (b) classes. *n* = 10. PE, phosphatidylethanolamine; LPE, lysophosphatidylethanolamine. The transparency of each bar is proportional to the significance. (c) Volcano plot showing the top 20 most different lipids after HT. (d and e) Effect of HT on Cer (d) and sphingolipid (e) content. Up: |log_2_(fold change)| > 1, down: |log_2_(fold change)| < −1, only the data of *P* < 0.05 are shown. *n* = 10. (f) HT affects the sphingolipid metabolism. (g) WB analysis of ASAH1 and ASAH2 in the SMT. (h) WB analysis of AKT phosphorylation in the SMT of DIO mice under HT conditions with or without NOE treatment.

Specifically, HT improved the composition of various fatty acyls of PEs and lyso-PEs (LPEs) in the SMT of DIO mice (Fig. 7a and b). Further analysis of the top 20 DLs in the SMT of obese mice after HT revealed a decrease in Cers, including Cer (22:1;2O/38:6;O), Cer (20:0;2O/38:5), and Cer (12:0;3O/36:0;(2OH)). On the other hand, sphingolipids, such as SM (52:8;2O), Cer (12:2;2O/30:6;O), SM (12:1;2O/36:0), Cer (20:3;2O/38:6;O), SM (34:6;3O), and SM (34:0;2O), were upregulated after HT (Fig. 7c). Importantly, HT decreased Cer levels while increasing SM and PE levels in the SMT of DIO mice (Figs. 6f, 7d and e). Cers can be metabolized to generate SM and PE. The analysis of lipidomic data revealed that HT promoted the breakdown of Cer into PE and SM in the SMT of DIO mice (Fig. 7f). Therefore, these findings supported the notion that HT promoted Cer breakdown into PE and SM in the SMT of DIO mice, potentially contributing to the improvement of IR.

Spearman’s correlation analysis between the DEGs and the DLs further revealed associations between HT-induced genes and sphingolipid metabolism (Supplementary Fig. S6). Upregulated genes (*Hspa1a*, *Hspa8*, *FoxO1*, *Gadd45g*, *Slc27a3*, *Ppp1r3c*, and *Cdkn1a*) were positively associated with upregulated sphingolipids and negatively associated with downregulated Cers in the SMT of DIO mice. Conversely, downregulated genes (*Pck1*, *Socs2*, *Socs3*, *Pygl*, *Pfkfb3*, *Vcam1*, and *Mmp9*) were negatively associated with downregulated Cers and positively associated with upregulated SMs in the SMT of DIO mice. These correlations further supported the role of HT in promoting Cer catabolism in the SMT of DIO mice (Supplementary Fig. S6).

Considering the role of ceramidase in Cer metabolism, we were curious about the impact of HT on ceramidase levels. We examined the acid ceramidase N-acylsphingosine amidohydrolase 1 (ASAH1) and neutral ceramidase ASAH2 through WB analysis and found that HT did not affect the expression of ASAH2 (Fig. 7g). Furthermore, HT did not alter the protein level of ASAH1 in normal mice, but it significantly increased the ASAH1 level in obese mice (Fig. 7g). This observation suggested that ASAH1 may be crucial for HT-induced Cer breakdown and may contribute to enhancing insulin sensitivity. To confirm the role of ASAH1 in improving insulin sensitivity, we used N-oleoylethanolamine (NOE), an ASAH1 inhibitor, to inhibit ASAH1 in the SMT of DIO mice and monitored the phosphorylation level of AKT473 and AKT308 after HT for 4 h. The results indicated that inhibition of ASAH1 significantly suppressed the phosphorylation levels of AKT473 and AKT308 (Fig. 7h), underscoring the essential role of ASAH1-mediated Cer breakdown in enhancing insulin sensitivity of SMT in DIO mice. In summary, these findings suggested that HT promotes Cer breakdown in the SMT of DIO mice, thereby contributing to the improvement of insulin sensitivity.

### HT enhances the components of Cers and cholesteryl esters (CEs) in LDs of skeletal muscle cells of DIO mice

**Figure 8:**
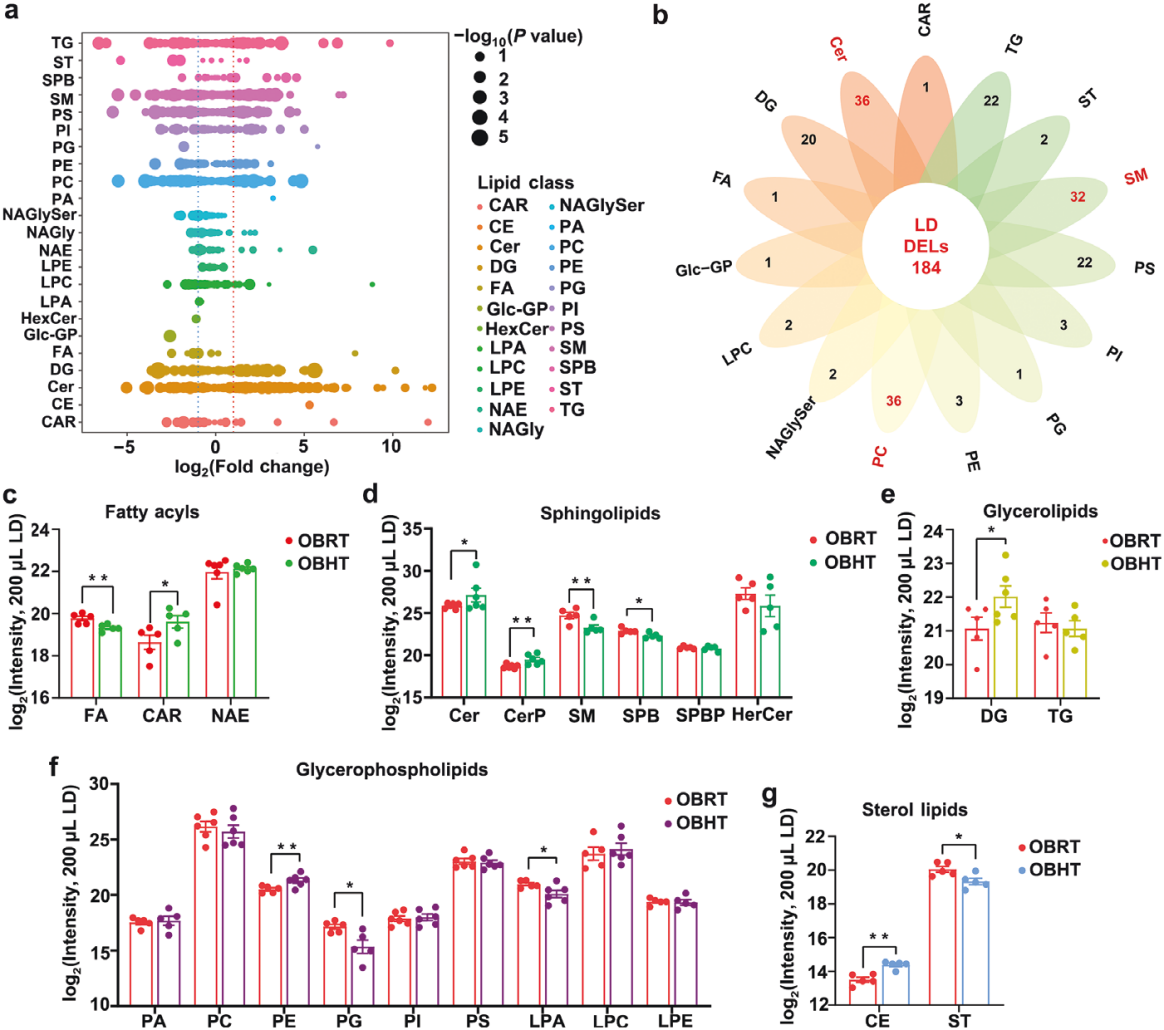
HT alters the lipid composition of LDs in the SMT of DIO mice. (a) The log_2_(fold change) values in lipid class in LDs of OBRT vs OBHT group; the significance values are displayed as − log_10_(*P* value). (b) Flower plot showing the changes in all lipids of LDs in skeletal muscle cells of DIO mice after HT. (c−g) The fold changes in fatty acid acyl chains (c), sphingolipids (d), glycerolipids (e), glycerophospholipids (f), and sterol lipids (g) in LDs of the SMT in obese mice. CAR, acyl carnitine; CE, cholesteryl ester; CerP, Cer phosphate; NAE, N-acyl ethanolamines. Data are presented as means ± SEM, *n* = 10. ^*^P < 0.05, ^**^P < 0.01.

Given that LDs are organelles involved in stress resistance, immune regulation, and signal transduction [25, 26], LDs might participate in alleviating heat stress damage and IR in the SMT of DIO mice. We used ultra performance liquid chromatography tandem mass spectrometry (UPLC-MS/MS) to determine the lipid composition of LDs in skeletal muscle cells and identified 8041 fragmental ions (Supplementary Table S6). Using the LIPIDMAPS and LipidBlast databases for characterization, a total of 1300 lipids were annotated, including 263 Cers, 199 SMs, 192 PCs, 141 PSs, 117 DGs, 97 TAGs, and other lipids (Supplementary Table S7). Subsequently, using *P* < 0.05 and |log_2_(fold change)| > 1 as the criteria for DLs, we identified 184 DLs in the LDs, the top three lipid classes of which were Cers, PCs, and SMs (Fig. 8a and b). We classified all lipids to determine which lipid classes were affected by HT in the LDs of the skeletal muscle cells of DIO mice. It was found that HT significantly affected the sphingolipid composition of LDs, as it increased the Cer and Cer phosphate (CerP) content and decreased the SM and SPB content in the skeletal muscle cells of DIO mice (Fig. 8d). HT induced an increase in acyl carnitines (CARs) and DGs and an decrease in fatty acyls in LDs of the obese mice (Fig. 8c and e). Furthermore, HT affected glycerophospholipid metabolism by increasing PE content and decreasing prostaglandin (PG) and lysophosphatidic acid (LPA) levels (Fig. 8f). Given the variations in CE and ST content, it was speculated that HT probably promoted sterol esterification in the LDs of skeletal muscle cells of obese mice (Fig. 8g). In summary, HT-induced accumulation of Cers and CEs in LDs not only alleviates the lipotoxicity of excess Cers and CEs in skeletal muscle cells but also improves their adaptability to hyperthermic environments in DIO mice.

## Discussion

In this study, a multi-omics analysis was conducted to investigate the effects of HT on SMT in DIO mice. The results revealed several beneficial effects of HT on SMT metabolism and function. One of the key findings was the improvement in SMT carbohydrate and lipid metabolism gene expression. HT activated sphingolipid metabolism and Cer catabolism in an HSF1-dependent manner. This activation resulted in the promotion of myogenic glycogen synthesis and lipolysis and ultimately improved insulin sensitivity in the SMT of DIO mice. Interestingly, it was observed that the SMT of DIO mice was more resistant to HT-induced injury compared to normal mice. This resilience could be attributed to the increased production of 5-hydroxytryptamine and γ-glutamylcysteine, which enhanced the antioxidant capacity of the SMT in DIO mice. HT may have stimulated the expression of antioxidant genes in the SMT, similar to its effect on brown adipose tissue as previously observed [27]. The induction of antioxidant genes in the SMT could protect against heat stress injury and contribute to the improvement of obesity in DIO mice.

Reduced glucose uptake by skeletal muscle is one of the mechanisms leading to the development of IR [3]. In our study, we observed that HT affected glucolipid metabolic signaling pathways, such as PI3K-AKT and FoxO signaling pathways, promoting the expression of glycogen synthesis-related gene *Ppp1r3c*, while inhibiting the expression of glycogen catabolism-related genes *Socs2* and *Socs3*. As a result, serum glucose levels decreased and myoglycogen levels increased, indicating that HT enhanced glucose uptake by the SMT in DIO mice, thus facilitating glucose storage. Furthermore, HT directly targeted skeletal muscle and promoted glucose transport by increasing GLUT4 protein expression, thereby improving obesity and IR [28]. Our in-depth analysis revealed that the improved glucolipid metabolism induced by HT was largely attributed to the maintenance of the decay phase of HSF1. Cells regulate their stress response by activating *Hsf1* transcription, which mediates the expression of Hsp70 and other molecular chaperone proteins [29, 30]. When exposed to ambient temperature and nutrient stress, serine residues 303 and 307 of HSF1 are phosphorylated during the attenuation phase [23, 31]. In our study, HT enhanced the protein levels of phosphorylated HSF1 (p-Ser303) and its interaction with HSP70 in the SMT of DIO mice, ultimately leading to high levels of HSP proteins in cells [23]. Interestingly, HSP70 has also been reported to alleviate IR in obese and diabetic patients [17]. Furthermore, sensing local mild thermal effects and activating thermogenesis through HSF1, beige fat can effectively resist and treat obesity, as well as ameliorate metabolic disorders such as IR and hepatic lipid deposition [32]. These results suggest that targeting HSF1 may be an effective strategy for the treatment of obesity and IR.

Ultimately, HT altered the lipid composition of SMT in DIO mice by affecting signaling pathways involved in carbohydrate and lipid metabolism. We observed a significant increase in DG content following HT. It is worth noting that previous evidence suggest that elevated DG levels are associated with impaired insulin signaling and IR [33]. However, our findings contradict this notion. Interestingly, athletes have higher DG content and more LDs in skeletal muscle, yet they possess higher insulin sensitivity. This difference in insulin sensitivity is thought to be influenced by the subcellular localization of DG, whether it is membrane bound or part of neutral LDs [34]. Consistent with these observations, our results indicated that HT induced the accumulation of DG specifically in LDs, which may alleviate the impairment of insulin signaling through DG activation of protein kinase C isoforms [35]. Furthermore, HT enhanced the expression of FoxO1, a transcription factor that plays a role in regulating the insulin signaling pathway in mammals [36]. This finding suggested that HT activates the insulin signaling pathway in the SMT of DIO mice, thus triggering the reprogramming of glucolipid metabolism in SMT of DIO mice.

HT activated sphingolipid metabolism in the SMT of DIO mice, which is associated with cell signaling and the development of several diseases, including diabetes and atherosclerosis [14]. Complex sphingolipids are formed by attaching various head groups to Cer or dihydroglyceramide backbone [37]. However, numerous studies have demonstrated that Cer accumulation plays a significant role in IR and cardiovascular disease [38]. In our study, HT increased Cer breakdown in the SMT of DIO mice, which could be crucial for ameliorating DIO-induced IR since Cer antagonizes insulin signaling at the level of RACα serine/threonine protein kinase (also known as AKT or protein kinase B) [39]. Reduced Cer accumulation significantly enhanced insulin sensitivity in DIO mice [40]. The breakdown of Cers increased SM, potentially attributed to the HT-induced expression of ASAH1 in the SMT of DIO mice. Importantly, the inhibition of ASAH1 mitigated HT-relieved IR in the SMT of DIO mice. Consistent with these findings, the overexpression of acid ceramidase prevented saturated fatty acids from impairing insulin action in cultured C2C12 myotubes. Furthermore, the overexpression of acid ceramidase in the liver improved hepatic insulin sensitivity. These results collectively suggest an essential role for acid ceramidase in insulin action. Additionally, Cer catabolism also led to an increase in SM, and the exact mechanism of this phenomenon needs further exploration. Moreover, Cers can induce HT-induced apoptosis by post-transcriptionally regulating the inhibition of anti-apoptotic HSP70 [41]. Therefore, Cer breakdown contributed to maintaining HSP70 levels, which may be another reason why the SMT in DIO mice was protected against heat stress injury.

Interestingly, HT promoted the synthesis and accumulation of Cers in the LDs of skeletal muscle cells, suggesting that LDs may enhance adaptation to heat stress in DIO mice by sequestering harmful lipids. Consistent with this, Cers can be converted to acyl Cers and stored in LDs, thereby reducing Cer levels in the cytoplasm. This implies that promoting Cer storage in LDs could be a novel strategy for obesity treatment. Further studies are required to elucidate the specific mechanisms underlying these effects and to explore their potential therapeutic applications in obesity and metabolic disorders.

**Figure 9:**
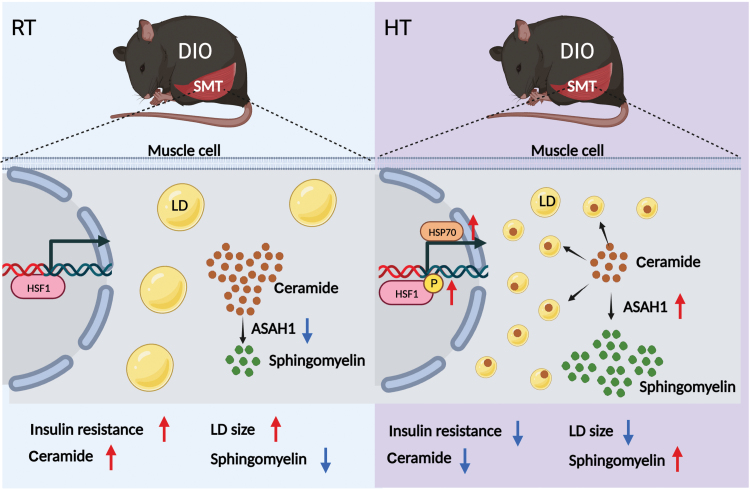
Schematic illustration of high temperature ameliorating HFD-induced IR in the SMT of DIO mice. RT, room temperature; HT, high temperature (40°C, 4 h); DIO, diet-induced obesity; HSF1, heat shock factor 1; HSP70, heat shock protein 70; LD, lipid droplet; ASAH1, acid ceramidase N-acylsphingosine amidohydrolase 1.

In conclusion, the findings presented in our study provide valuable insights into the role of HT in modulating sphingolipid metabolism as well as insulin signaling in the SMT of DIO mice (Fig. 9). However, a more comprehensive understanding of the molecular pathways involved and the long-term effects of HT is necessary. Future research should aim to investigate the downstream signaling events and molecular interactions involved in HT-induced changes in glucolipid and sphingolipid metabolism. Additionally, it would be beneficial to explore the effects of HT in different animal models and human subjects to validate the findings and assess the potential translational applications. Furthermore, it would be valuable to examine the duration and timing of HT to maximize its beneficial effects on obesity and metabolic disorders. This would help in developing targeted therapeutic strategies and optimizing the clinical application of HT. While our study highlights the potential of HT as a promising therapeutic approach for obesity and metabolic disorders, further research is needed to unravel the underlying mechanisms and determine its efficacy, safety, and long-term effects.

## Materials and methods

### Experiment processing and sample collection

In this study, 3-week-old male C57BL6/J mice (10.0 ± 0.5 g) were divided into normal (*n* = 10) and obese (*n* = 60) groups. The two groups of mice were housed for 9 weeks as previously described [27]. Subsequently, mice in the obesity group were sub-divided into OBRT and OBHT groups (*n* = 25 per group) and housed at either 25°C or 40°C for 4 h in an artificial climate chamber (PRX-280A) [42, 43]. Then, one fasting blood sample was collected from each mouse, from which 200 uL without anticoagulant was used for glucose detection (GM1118, Wuhan Servicebio Technology). All the mice were anesthetized and later euthanized by cervical dislocation. SMT samples were collected for RNA, lipid, and metabolite extractions. In addition, fresh SMT was immediately tested for ROS level detecion (GDP1018, Wuhan Servicebio Technology). In a separate experiment, obese mice were treated for 2 weeks with vehicle or NOE (5 mg/kg body weight, *per os* (p.o.), once daily). All the mice were anesthetized and later euthanized by cervical dislocation. SMT samples were collected for RNA and protein extractions. Before the HT, all mice underwent a 12-h fasting period. During the HT, all mice had unrestricted access to water, but food was not provided.

### Histological analysis of SMT after high-temperature intervention

Hematoxylin and eosin staining and TEM were used to observe SMT morphology as previously described [27]. The markers involved in heat stress response (anti-HSP70 antibody, 1:500, 10995-1-AP, Proteintech), skeletal muscle cell apoptosis (anti-TNFR1 antibody, 1:200, 60192-1-Ig, Proteintech), and mitochondrial function (anti-CytoC antibody, 1:200, 66264-1-Ig, Proteintech) were examined using immunofluorescence analysis. To determine the effect of HT on myoglycogens, periodic acid-Schiff (PAS) staining and a myoglycogen test were performed on SMT. To visualize the effects of HT on lipid metabolism of the SMT in mice, TEM assay was performed. A field of view of the same cross-section was chosen to quantify the changes of LDs in the SMT (*n* > 3). The areas of LDs and myofibers were calculated separately using image-Pro plus 6.0 software, and their percentages were used to determine the changes in lipid composition after HT.

### Transcriptome sequencing analysis

Total RNA was isolated from the SMT of the OBRT and OBHT groups, and was used for library construction and sequencing to generate 150 bp paired-end reads. Raw data in fastq format were first processed to obtain clean data by removing the reads containing adapters, ploy-N > 10%, and other low-quality reads. The quality of sequencing data and the number of genes are shown in Supplementary Table S8. The genome and gene annotation files were used to build the index of the reference genome, and Hisat2 (v2.1.0) was used to align paired-end clean reads. The initial alignment information was obtained by analyzing the BAM files using Samtools (v1.9) software. Each mapped gene read was counted using HTSeq (v0.6.0). DEGs between the OBRT and OBHT groups were analyzed using the DESeq2 R package (1.20.0) [44]. Gene annotations and the KEGG pathway enrichment analysis were performed to determine the function of the DEGs [45, 46]. Finally, the DEGs were applied to gene–gene interaction network and clustering analysis using ggcor (v0.9.8) software and the STRING Database (v11.5.0).

### Metabolite extraction

A total of 50 mg frozen sample of the SMT (OBRT and OBHT groups) was transferred to a 2-mL centrifuge tube, and 400 µL of extraction solution (methanol:water = 4:1 (v:v)) and a mixture of internal standards were added. After homogenization, the tissue was ground at 50 Hz for 6 min (−10°C) using a high-throughput tissue homogenizer, followed by an ultrasonic disruptor at 40 kHz for 3 min (5°C). The samples were allowed to stand for 30 min at −20°C and then centrifuged at 13,000 r/min at 4°C for 15 min. Finally, the supernatant was transferred to an injection vial with internal inserts for analysis using UPLC-MS/MS. The internal standard mixture was previously described [27].

### Metabolomic analysis

Metabolite extracts were analyzed using a Waters G2-XS QTOF mass spectrometer integrated with a Waters ACQuityH-UPLC@CLASS System. The metabolites were separated using a linear gradient of solution A (water, 0.1% formic acid) and solution B (acetonitrile, 0.1% formic acid). The gradient (0.4 mL/min) was as follows: *T* = 0–1 min: 95% A; *T* = 1–9 min: 95% A–60% A; *T* = 9–9 min: 60%A–10%A; *T* = 19–21 min: 10%A–0%A; *T* = 21–26 min: 0% A; *T* = 26–30 min: 0% A–5% A. For the reverse phase, 10 µL metabolites were injected into an ACQUITY UPLC HSS T3 column (100 mm × 2.1 mm Column, 1.8 µm; Waters) and maintained at 45°C. The MS/MS data were acquired using MassLynx (V4.1), and Progenesis QI software was used for metabolomics data analysis based on the HMDB and KEGG databases. Normalized data were subjected to multivariate statistical analysis using an in-house metabolome script. Unsupervised principal component analysis (PCA) was used to observe the overall distribution among the samples, and OPLS-DA was used to distinguish the overall differences in metabolism among the groups. Finally, we used VIP > 1 and *P* < 0.05 as the standard to filter for DMs.

### LD isolation

The cytoplasmic components of skeletal muscle cells were originally divided by applying a cytoplasmic segregation kit, and the LDs from the cytoplasm were separated and purified as previously reported [47]. In brief, the upper LDs were withdrew by adding the cytoplasmic components to buffer A after ultracentrifugation (260,000 × g) at 4°C for 30 min. Washes of the LDs were performed three times by adding buffer B, gathering the upper liquid (Supplementary Fig. S7). A fraction obtained was applied to determine the lipid composition.

### Lipidomic analysis

Lipids were extracted from the SMT as previously described [27]. The lipid extracts were analyzed using a Waters G2-XS QTOF mass spectrometer integrated with a Waters ACQuityH-UPLC@CLASS System. Lipids were segregated via a linear gradient of solution, comprising solution A (40% acetonitrile, 0.1% formic acid, and 5 mmol/L ammonium formate) and solution B (1:9 acetonitrile/isopropanol, v/v, 0.1% formic acid, and 5 mmol/L ammonium acetate). The gradient (0.3 mL/min) was as follows: *T* = 0–4 min: 85% A; *T* = 4–5 min: 85% A–52% A; *T* = 5–22 min: 52%A–18%A; *T* = 22–23 min: 18%A–1%A; *T* = 23–24 min: 1% A; *T* = 24–30 min: 1% A–85% A. With a reversed phase, the lipids were shot onto a C18 column (2.1 mm × 100 mm, 1.7 μm) maintained at 45°C. The MS/MS data were acquired using MassLynx (V4.1), and the software Progenesis QI was used to analyze the lipidomics data based on the LIPIDMAPS database. To increase the accuracy of lipid identification, Mass Spectrometry-Data Independent Analysis (MS-DIAL) software were used to annotate the raw data based on the LipidBlast database. Finally, the identified lipids were combined for subsequent data analyses [48].

### WB analysis and CoIP assay

The proteins in the SMT were extracted with a strong and weak RIPA lysate (G2002 and G2033, respectively, Wuhan Servicebio Technology) for WB and CoIP assays, respectively. The extracted proteins were subjected to WB analysis to test the expression of the corresponding proteins (HSP70, 1:4000, 10995-1-AP, Proteintech; HSF1, 1:2000, 16107-1-AP, Proteintech; HSF1(p-Ser 303), 1:2000, TA3372, Abmart; AKT, 1:1000, AF6261, Affinity; AKT(p-Ser473), 1:1000, AF0016, Affinity; AKT(p-Thr308), 1:1000, AF3262, Affinity; ASAH1, 1:1000, 11274-1-AP, Proteintech; ASAH2, 1:1000, 27742-1-AP, Proteintech; GAPDH, 1:10000, 60004-1-Ig, Proteintech). In addition, a CoIP assay was performed to examine the interaction between HSF1 and HSP70 proteins. The detailed procedures were described in the previous publications [49].

### Cell culture and transfection

The C2C12 cells were grown in Dulbecco’s Modified Eagle Medium (DMEM; HyClone, Logan, UT, USA) with 10% fetal bovine serum (FBS; #SH30396.03, Hyclone, Canada), 1% penicillin/streptomycin in dishes at 37°C, in a humidified atmosphere with 5% CO_2_. For transfection, a total of 2 μL Lipofectamine® 2000 transfection reagent (11668-019, Thermo Fisher) diluted in 25 μL Opti-MEM (51985034, Thermo Fisher) was prepared. In addition, 1 μg plasmid was diluted with 25 μL Opti-MEM and incubated for 5 min at RT. The 50 μL mixture of Lipofectamine^®^ 2000 and plasmids was added into one well (24-well plate). After 5–6 h, the medium was renewed and the cells were incubated for 24–48 h for further use in the following experiments. All analyses were done with three biological replications (three wells of cells per replication).

### C2C12 differentiation

The C2C12 differentiation process followed the established protocol in our laboratory [49]. When the cells reached approximately 90% confluence, they were transferred to a differentiation medium (DMEM) containing 2% horse serum (Gibco) and cultured for 5–7 days. During this period, the medium was refreshed every two days.

### ROS level detection

ROS levels in the SMT were assessed using a ROS Assay Kit (#S0033, Beyotime) based on the 2ʹ,7ʹ-dichlorodihydrofluorescein diacetate (DCFH-DA) method. The ROS level was measured using a microplate spectrophotometer (PerkinElmer EnSpire, Waltham, MA, USA) with excitation and emission wavelengths set at 488 and 525 nm, respectively. ROSup, an activator to increase the ROS level in the SMT, was added to the slides with DMEM at a ratio of 1:1000 (v:v) and incubated at 37°C.

### Immunofluorescence assay

The paraffin-embedded sections were subjected to dewaxing by immersion in xylene for 20 min, repeated twice. Subsequently, the sections were treated with anhydrous ethanol for 5 min, repeated twice, followed by a 5-min immersion in 75% alcohol. After this, the sections were rinsed with distilled water for 3 min and finally washed with PBS for 5 min. Antigen retrieval was carried out by immersing the sections in a 20× Tris-EDTA antigen retrieval solution (pH = 8.0). The sections were heated in a microwave oven at medium heat for 8 min until boiling, and then allowed to cool for 8 min. Subsequently, the sections were microwaved at medium-low heat for 7 min, followed by cooling naturally for 20–30 min. The sections were rinsed three times with PBS for 5 min each time. Next, the sections were incubated with 3% BSA for 30 min at 37°C. A drop of the corresponding primary antibody (diluted 1:100 in 3% BSA) was placed on each section and incubated for 2 h at RT. The sections were then rinsed three times with PBS. A drop of the corresponding secondary antibody was placed on each section and incubated for 1 h at RT, followed by three PBS rinses. Each slide was incubated with a drop of DAPI (4ʹ,6-diamidino-2-phenylindole) for 15 min at RT and then rinsed three times with PBS. Finally, the sections were sealed with an anti-fluorescence mounting medium and immediately examined under a fluorescence microscope. The confocal laser scanning microscope (Carle Zeiss, German) was used to observe the slide of cells. The images were analyzed by ZEN pro software (Carle Zeiss, German), ImageJ, and Photoshop CS6 (Adobe).

### Correlational assay and statistical analysis

The correlational analysis method was as previously described [27] and their coefficients were calculated using the R (v4.0.5) program. For each biological indicator, the acquired data were presented as the mean ± standard deviation (SD), and GraphPad 8.0 was applied to visualize the data.

## Supplementary Material

loae012_suppl_Supplementary_Figures_S1-S7

loae012_suppl_Supplementary_Table_S2

loae012_suppl_Supplementary_Table_S3

loae012_suppl_Supplementary_Table_S4

loae012_suppl_Supplementary_Table_S5

loae012_suppl_Supplementary_Table_S6

loae012_suppl_Supplementary_Table_S7

loae012_suppl_Supplementary_Table_S8

loae012_suppl_Supplementary_Table_S1

## Data Availability

All study data are included in the article and/or supplementary information. Materials and reagents are available upon request.
