# Supplementary material for: High temperature ameliorates high-fat diet-induced obesity by promoting ceramide breakdown in skeletal muscle tissue
Source: Life Metab. 2024 Apr 3;3(5):loae012. doi: 10.1093/lifemeta/loae012 (PMC11749596; doi:10.1093/lifemeta/loae012)
Supplement: loae012_suppl_Supplementary_Figures_S1-S7 [file loae012_suppl_Supplementary_Figures_S1-S7.docx]

### High temperature ameliorates high-fat diet-induced obesity by promoting ceramide breakdown in skeletal muscle tissue

**Qiankun Wang****^1,4,‡^, Lupeng Chen^1^****^,‡^, Junzhi Zhang^1^, Yue Liu^1^, Yi Jin^1^,** **Jian Wu^1^,** **Zhuqing Ren^1,2,3,*^**

**Supplementary material Figure S1**


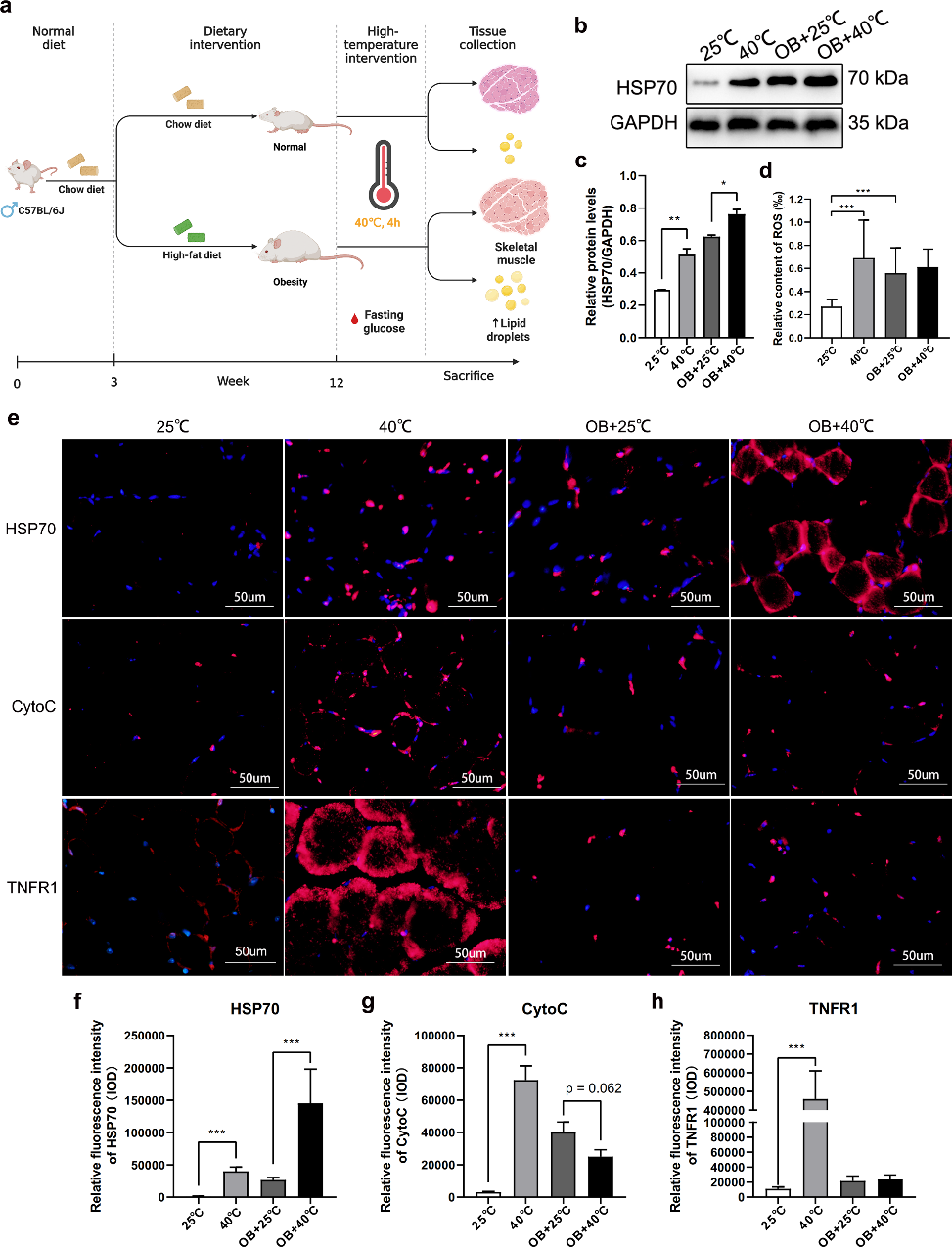


**Supplementary Figure S1** A comparative analysis of the effects of heat treatment on SMT in normal and obese mice. (a) Experiment flow of heat treatment on normal and obese mice. (b and c) Representative bands (b) and protein levels (c) of HSP70 between normal and obese mice after heat treatment. *n* = 3. ^*^*P* < 0.05, ^**^*P* < 0.01. (d) Effect of heat treatment on ROS levels in the SMT of normal and obese mice. *n* = 3. ^*^*P* < 0.05, ^**^*P* < 0.01. (e) Expression changes of HSP70, CytoC, and TNFR1 protein by immunofluorescence analysis in mouse SMT. Notes: The first row is the confocal images of SMT sections for the HSP70, the middle row is CytoC, and the bottom row is TNFR1 in 25℃, 40℃, OB + 25℃, and OB + 40℃ groups (× 400). Blue: DAPI; Red: HSP70/CytoC/TNFR1. (f−h) The statistical analysis of positive signal intensity for HSP70 (f), CytoC (g), and TNFR1 (h) immunofluorescence staining. *n* = 3. ^***^*P* < 0.001.

**Supplementary material Figure S2.**


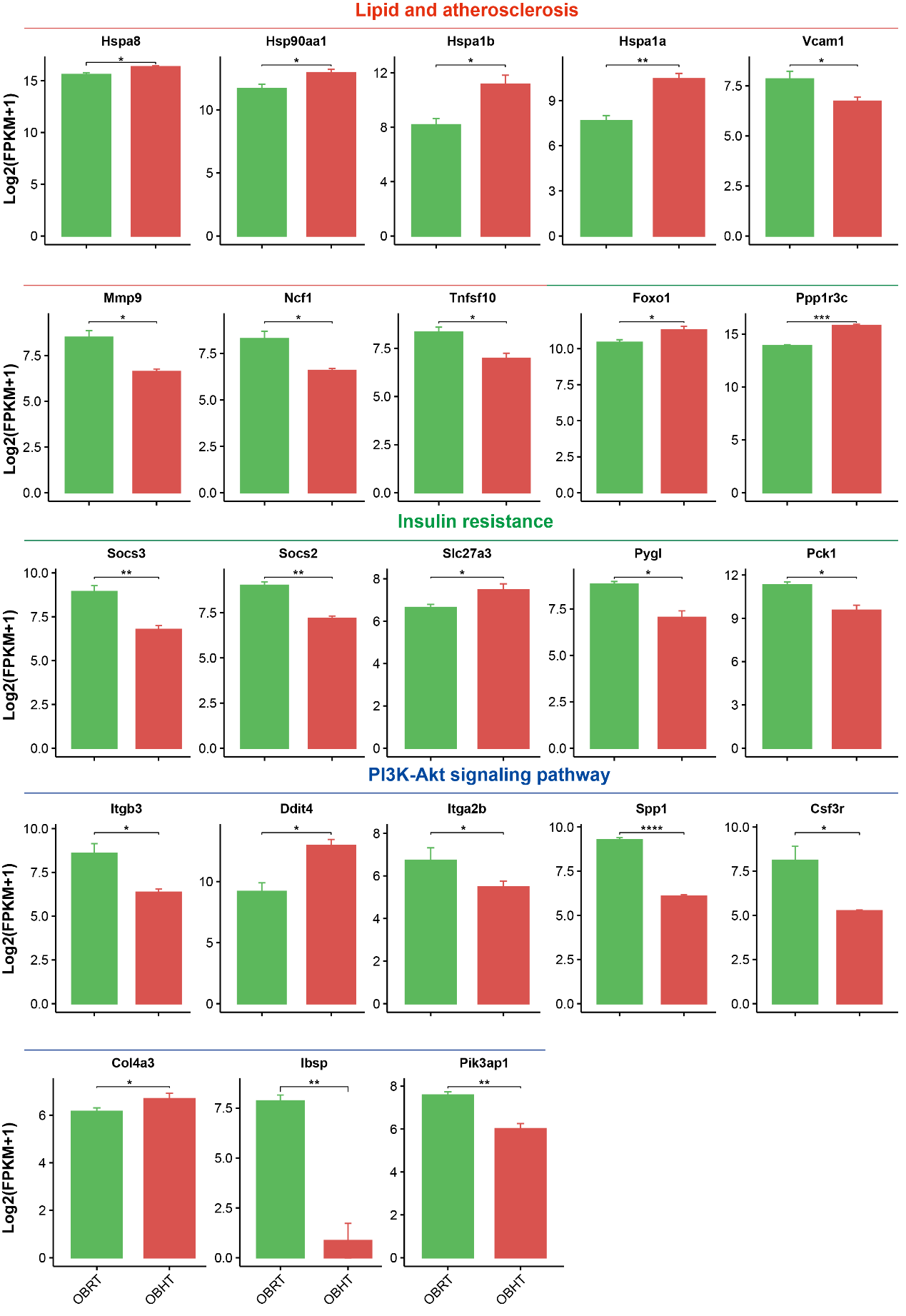


**Supplementary Figure S2** The expression of relevant genes in SMT of DIO mice in transcriptome data. FPKM, fragments per kilobase of exon model per million mapped fragments. *n* = 10. ^*^*P* < 0.05, ^**^*P* < 0.01, ^***^*P* < 0.001, ^****^*P* < 0.00001.

**Supplementary material Figure S3.**


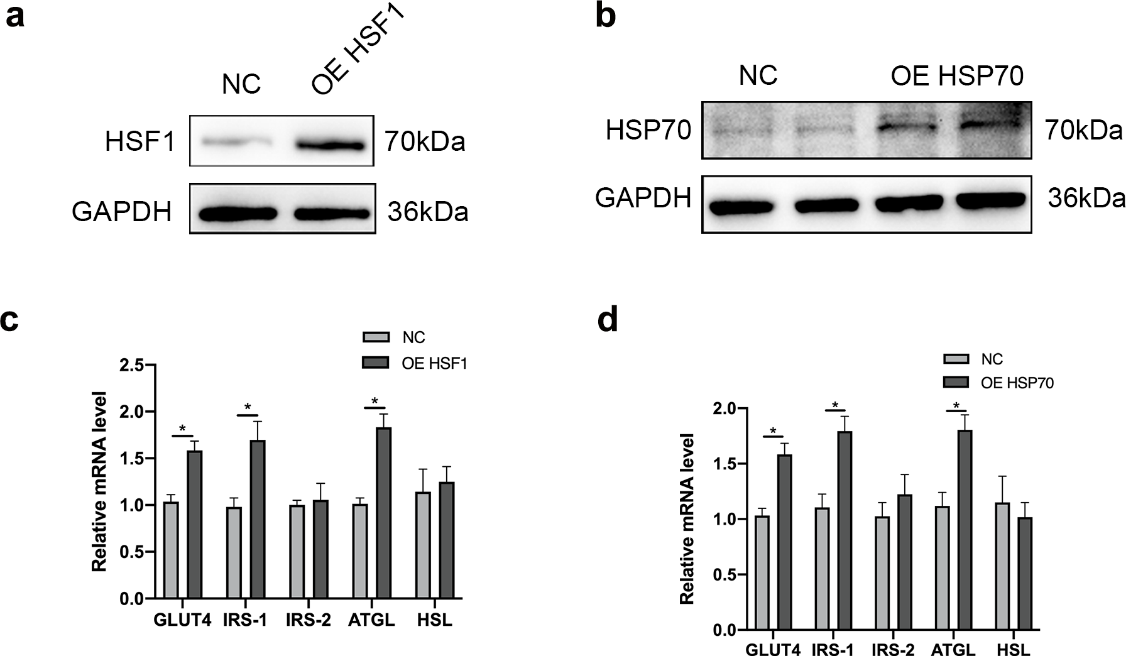


**Supplementary Figure S3** Heat treatment kept the attenuation phase of HSF1 in SMT of DIO mice. (a and b) Western blot detection of HSF1 (a) and HSP70 (b) protein overexpression. (c and d) The mRNA expression of carbohydrate and lipid metabolism after HSF1 (c) and HSP70 (d) protein overexpression. *n* = 3. ^*^*P* < 0.05.

**Supplementary material Figure S4.**


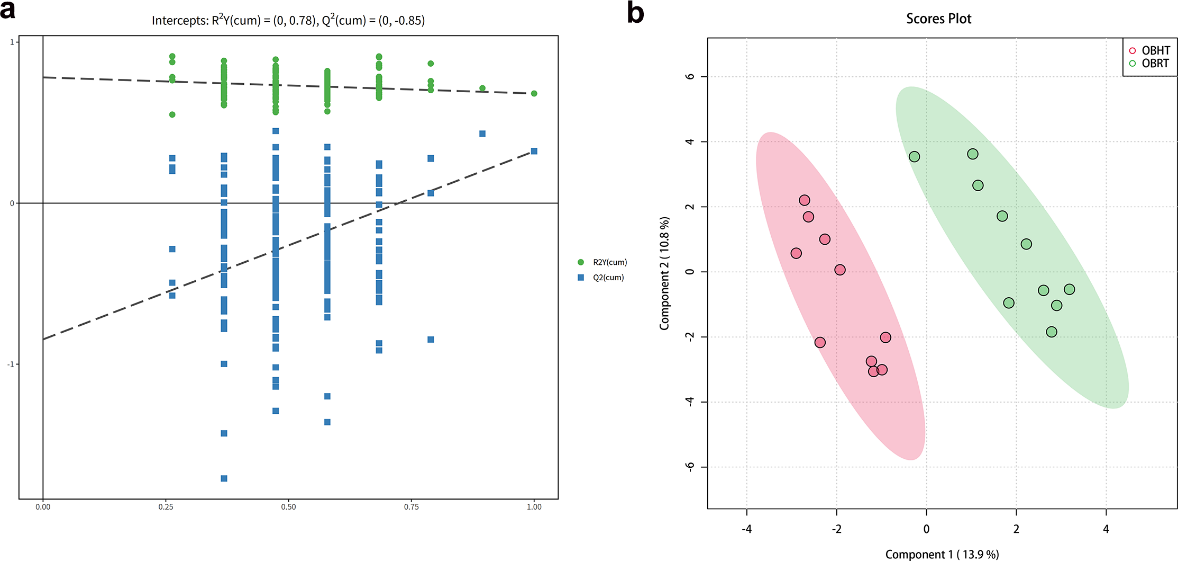


**Supplementary Figure S4** OPLS-DA analysis of metabolomic data in the SMT of obese mice. (a) OPLS-DA models for all metabolites in the SMT of DIO mice. (b) Supervised OPLS-DA for all samples.

**Supplementary material Figure S5.**


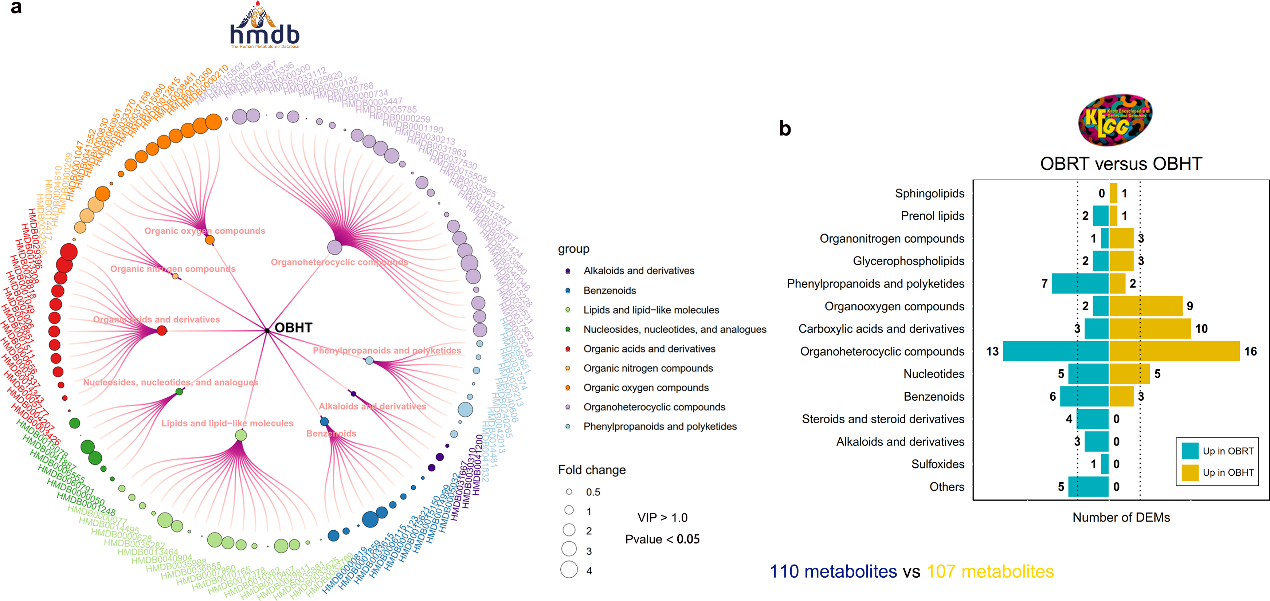


**Supplementary Figure S5** Screening and identification of differential metabolites in the SMT of obese mice. (a) Characterization and classification for HMDB database. (b) Characterization and classification for KEGG database. VIP, variable important in projection; HMDB, Human Metabolome Database; KEGG, Kyoto Encyclopedia of Gene and Genomes (KEGG) COMPOUND Database.

**Supplementary material Figure S6.**


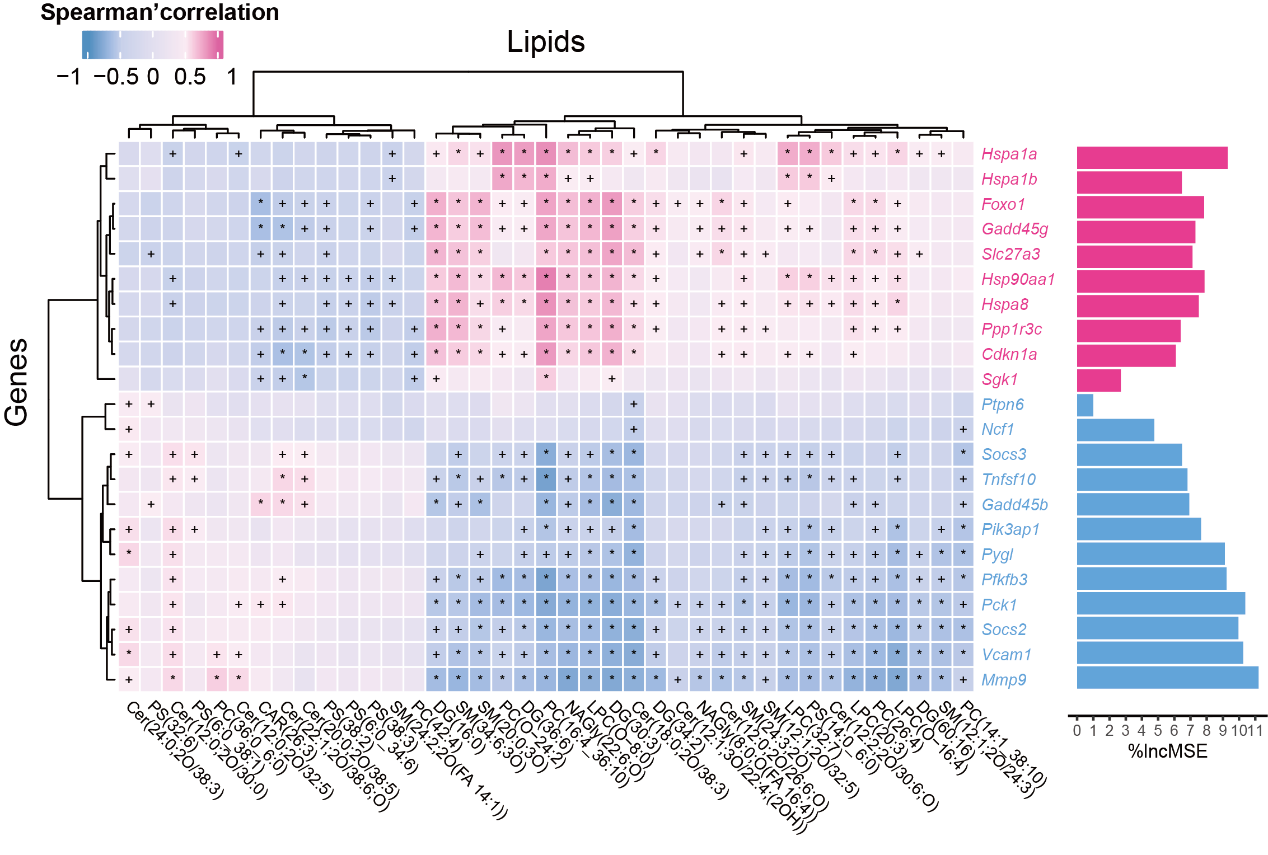


**Supplementary Figure S6** The correlation analysis between differentially expressed genes and differential lipids in SMT of obese mice. Red indicates positive correlation between gene and lipid, and blue indicates negative correlation between gene and lipid. Red genes areupregulated and blue gene aredownregulated. +*P* < 0.05, ^*^*P* < 0.0.1. The importance of genes was determined based on the percentage increase in mean square error (%IncMSE) in predicting changes in lipid content when a random replacement was made for the expression of each gene.

**Supplementary material Figure S7.**


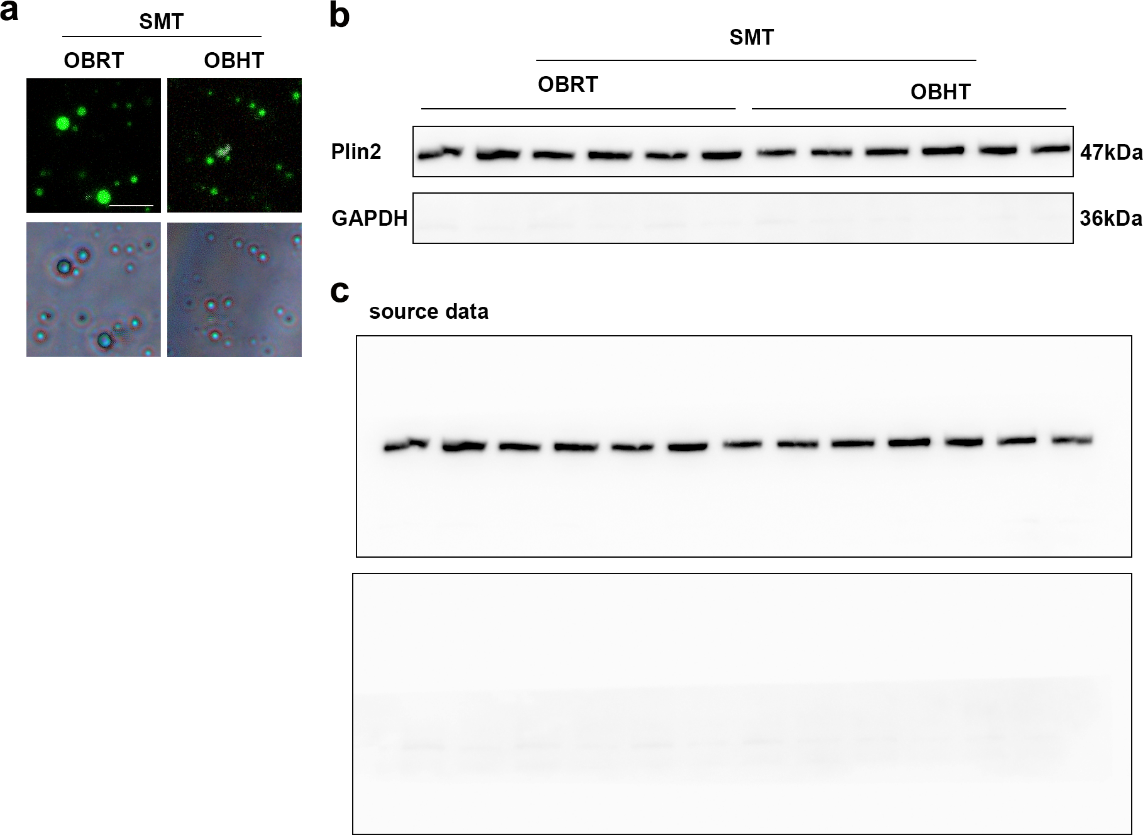


**Supplementary Figure S7** Morphology and purity assay of lipid droplets isolated from the SMT of DIO mice. (a) Confocal images of purified LD fractions from the SMT of DIO mice. Scale bar = 20 μm. (b and c) Detection of the marker protein perilipin 2 (Plin2) in isolated LDs.
