# Supplementary material for: High temperature ameliorates high-fat diet-induced obesity by promoting ceramide breakdown in skeletal muscle tissue
Source: Life Metab. 2024 Apr 3;3(5):loae012. doi: 10.1093/lifemeta/loae012 (PMC11749596; doi:10.1093/lifemeta/loae012)
Supplement: loae012_suppl_Supplementary_Table_S8 [file loae012_suppl_Supplementary_Table_S8.docx]

**Supplementary** **Table S8 SMT sequencing data quality and the number of genes.**

| Sampel | Clean read | Clean data | N(%) | Q20(%) | Q30(%) | GC(%) |
| --- | --- | --- | --- | --- | --- | --- |
| OBNC-1 | 55,799,857 | 16.74G | 0.01 | 97.51 | 93.77 | 49.01 |
| OBNC-2 | 46,697,903 | 14.01G | 0.01 | 97.31 | 93.23 | 48.78 |
| OBNC-3 | 46,853,781 | 14.06G | 0.01 | 97.52 | 93.70 | 48.97 |
| OBHT-1 | 41,695,249 | 12.51G | 0.01 | 97.70 | 94.11 | 49.55 |
| OBHT-2 | 37,617,100 | 11.27G | 0.01 | 97.08 | 93.01 | 49.18 |
| OBHT-3 | 42,715,788 | 12..81G | 0.01 | 97.64 | 93.86 | 49.72 |

N%: percentage of the fuzzy base; Q20: recognition accuracy rate of over 99% of the base; and Q30: recognition accuracy rate of over 99.9% of the base.
